# Portable all-in-one automated microfluidic system (PAMICON) with 3D-printed chip using novel fluid control mechanism

**DOI:** 10.1038/s41598-021-98655-9

**Published:** 2021-09-28

**Authors:** Yushen Zhang, Tsun-Ming Tseng, Ulf Schlichtmann

**Affiliations:** Chair of Electronic Design Automation, Technical University of Munich, 80333 Munich, Germany

**Keywords:** Lab-on-a-chip, Biomedical engineering

## Abstract

State-of-the-art microfluidic systems rely on relatively expensive and bulky off-chip infrastructures. The core of a system—the microfluidic chip—requires a clean room and dedicated skills to be fabricated. Thus, state-of-the-art microfluidic systems are barely accessible, especially for the do-it-yourself (DIY) community or enthusiasts. Recent emerging technology—3D-printing—has shown promise to fabricate microfluidic chips more simply, but the resulting chip is mainly hardened and single-layered and can hardly replace the state-of-the-art Polydimethylsiloxane (PDMS) chip. There exists no convenient fluidic control mechanism yet suitable for the hardened single-layered chip, and particularly, the hardened single-layered chip cannot replicate the pneumatic valve—an essential actuator for automatically controlled microfluidics. Instead, 3D-printable non-pneumatic or manually actuated valve designs are reported, but their application is limited. Here, we present a low-cost accessible all-in-one portable microfluidic system, which uses an easy-to-print single-layered 3D-printed microfluidic chip along with a novel active control mechanism for fluids to enable more applications. This active control mechanism is based on air or gas interception and can, e.g., block, direct, and transport fluid. As a demonstration, we show the system can automatically control the fluid in microfluidic chips, which we designed and printed with a consumer-grade 3D-printer. The system is comparably compact and can automatically perform user-programmed experiments. All operations can be done directly on the system with no additional host device required. This work could support the spread of low budget accessible microfluidic systems as portable, usable on-the-go devices and increase the application field of 3D-printed microfluidic devices.

## Introduction

During the past few decades, microfluidics has become more and more important in the fields of biology^1,2^ and chemistry^3^. This is due to its precise fluid control, using small fluid volumes and achieving high-throughput^4^. Microfluidics vastly reduces the size of laboratory experiments manipulating and controlling fluids to a range of microliters. It emerges as a powerful tool in many ways. An essential goal of this technology is to realize lab-on-a-chip systems, in other words, to miniaturize conventional experimental and analytical instruments into a chip format^5^. The state-of-the-art lab-on-a-chip is an ecosystem combined with an industrially fabricated microfluidic chip and the off-chip equipment to operate the chip. The advent of soft lithography was groundbreaking, enabling microfluidic technology and chip fabrication. It becomes the most widely used fabrication method. Still, photolithography requirements—from clean rooms^6,7^ to technical knowledge and skill^8,9^ for silicon processing—always present a substantial obstacle^10^ and are beyond the capability of many biology and chemistry scientists. Thus, conventionally, lab-on-a-chip users can only let companies or special facilities produce their desired chip. This fabrication technology makes the microfluidic chip far from accessible.

To operate the chip, most lab-on-a-chip systems heavily rely on bulky off-chip infrastructures such as compressed pressure sources, syringe pumps, and desktop computers to achieve controlled fluid manipulation functions. These infrastructures are not only relatively expensive but also not handy and accessible^11^. Their bulky size severely limits the applicability of such systems for critical applications, especially outdoors, such as point-of-care diagnostics, environmental monitoring, and bioterrorism detection^12^.

Therefore, there are currently multiple problems: (1) expensive, (2) bulky and inconvenient off-chip infrastructure, and (3) difficulties to manufacture the microfluidic chip. These problems have restricted the spread of lab-on-a-chip development both in the DIY community but also in the educational field. Thus, the need for an affordable and portable microfluidic system, and also an accessible chip fabrication method, increases.

Considering the price and the inconvenience of the off-chip infrastructures, Watson et al. recently developed an automated microfluidic control system using pump and solenoid valves controlled by microcontrollers^13^. Further, a similar design was proposed by Li et al., where also microcontrollers are used to control a miniature DC diaphragm pump and small solenoid valves^12^. Both of the proposed designs of handheld or portable microfluidic control systems reduced the size of certain off-chip infrastructures and are applicable to drive elastic PDMS-membrane microfluidics or pressure-driven microfluidics. Though, to operate these systems, a host device, such as a computer or smartphone, is required to send operations to the system, while the user needs to operate manually on the host device to, e.g., control each pressure source one-by-one.

The requirements for clean room and dedicated silicon processing skills have limited the accessibility of conventional microfluidic chip fabrication. A recently emerging fabrication method—additive manufacturing or 3D-printing^14^—represents a much more convenient choice for the microfluidic chip production. Especially a consumer-grade photo-curing 3D-printer, which costs only a fraction of the cost of professional fabrication devices, can already provide very high-resolution printing up to an accuracy of a few µm^15–17^. While 3D-printing has shown promise in reducing cost and increasing ease of device accessibility, most of the 3D-printed devices, because of the printing material properties^16^, are mainly hardened, single-layered, inflexible and pressure-driven. Notably, the inflexible hardened single-layered chip cannot replicate the Quake-style^18^ (shape-changing elastic pneumatic) valve^16,19,20^—an essential actuator for automated controlled microfluidics. It is hardly possible to produce soft and flexible parts directly using photo-curing 3D-printer. Instead, Bonyár et al.^21^ and Chan et al.^22^ have designed 3D-printable valves, including a torque-actuated valve, rotary valve, and pushing valve, which can be operated manually to disrupt the flow. Manually actuated valves are not suitable for automated control. Recently, Keating et al.^19^ reported an MJM (Multi-Jet Modeling) 3D-printed assembled pneumatically controlled valve using multiple printing materials. Also, Au et al.^14^ have reported a fully 3D-printed, but comparatively large pneumatical valve. Finally, Lee et al.^20^ have recently shown a Quake-style 3D-printed valve using resin mixed with different chemicals with special procedures. Although these are possible 3D-printed valve designs, which act as conventional elastic membrane valves, they are either too large to be embedded into a small chip design or require dedicated skill to operate the printing and assembly process. Though, in general, 3D-printing has lowered the threshold of microfluidic chip fabrication, a suitable control mechanism comparable to valve-based-control remains elusive.

In this paper, we present an accessible standalone automated all-in-one portable microfluidic system—Pamicon, including the proposal of a novel way of controlling fluids inside a single-layer chip, easily fabricated with an accessible technique—photo-curing 3D-printing, without implementing any of the existing 3D-printable valve designs. The acronym Pamicon is an abbreviation for our work’s title: **P**ortable **A**ll-in-one automated **MI**crofluidic system with 3D-printed chip using novel fluid **CON**trol mechanism. While a state-of-the-art microfluidic system mainly includes expensive and bulky control devices and equipment, our system employs the concept of using inexpensive and conveniently accessible parts, which are particularly lightweight and easy to build. In addition, our system is based on a single board computer and thus no additional host device, such as a computer, is required to operate. This microfluidic system is assembled in a small compact size and is designed to work with 3D-printed chips. Our proposed way of controlling fluids may replace the need for conventional valves in individual experiments and can be used in more complex integrated microfluidic designs. This portable microfluidic system provides on-board regulatable pressure and vacuum generation and control by using multiple miniature DC peristaltic pumps, a single-board computer with a desktop-grade operating system and a touchscreen for user interaction. To emphasize the characteristics of all-in-one, our system provides multiple peripheral connectivity options, such as USB or Ethernet connection. They can be used to integrate additional sensors or devices. Our system is also loaded with a simple, intuitive graphical user interface for chip control and experiment design, which can be operated directly via the touchscreen. While our system has a desktop-grade operating system, users can also design and program their application-specific software in their preferred programming language to control the system. Even for the connection between the control system and the 3D-printed chip, we used accessible medical tubing sets (incl. needles) as connectors. The main contribution of the paper is two-fold. First, it shows a relatively cheap and accessible all-in-one portable microfluidic system design, which works without a host device and is extendible for in-place analysis. Second, it introduces a novel fluid-control mechanism, designed for single-layered 3D-printed microfluidic chips. We hope this work can help the spread of a low budget accessible microfluidic system as a portable, usable on-the-go device and increase the application field of 3D-printed microfluidic devices.

## Results

We have demonstrated the operation of this all-in-one portable microfluidic system with 3D-printed single-layer chips using our proposed control mechanism. The system provides up to 4 pressure/vacuum sources (pumps) with up to ± 7.3 psi for controlling the fluid inside the chip. The number of pumps can be increased easily without additional software effort. The dimension of the resulting system is 18 $\times$ 12 $\times$ 5.5 cm with a total weight of 446 g, and the total build cost is about €340.

**Figure 1:**
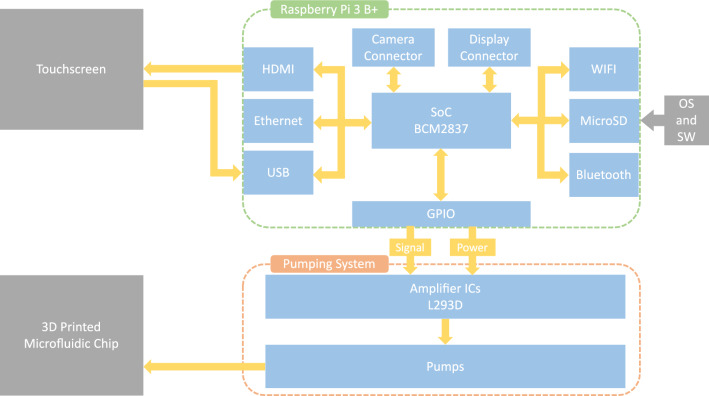
Block diagram of the system. The system consists of a touchscreen, a single board computer with MicroSD card as mass storage, and a pumping system capable of generating controllable positive and negative pressure output. The system can, for example, directly control a 3D-printed microfluidic chip.

The overall all-in-one portable system consists of 6 parts: (1) a single-board computer (SBC)—Raspberry Pi 3 B+ and a 5 V power supply; (2) a 7-in. touchscreen; (3) multiple DC miniature peristaltic pumps; (4) multiple signal amplifier ICs (Integrated Circuit) to drive the pumps; (5) intuitive programming software for controlling the pumps, which can be operated via the touchscreen; and (6) a 3D-printed microfluidic chip. The system can be controlled with our programming software directly on the system by using the touchscreen to input a sequence of operations. Figure 1 depicts the block diagram of the system.

The 3D-printing method is an accessible technique for microfluidic chip fabrication. Comparing different 3D-printing methods, DLP-SLA (digital light processing-stereolithography) resin 3D-printing method is the most accurate and precise 3D-printing process and is used commonly to fabricate microfluidic devices^15,16^. In contrast to multilayer soft lithography, the standard resin 3D-printing process results in a hardened, non-flexible object. Therefore, a chip with integrated valves like in PDMS microfluidic chips is not feasible with the usual printing materials and direct 3D-printing technique. Conventionally, a 3D-printed chip is pressure-driven, with no active flow control possible. In this work, we present a novel flow control mechanism for single-layer, especially 3D-printed, microfluidic chips.

### Fluid control mechanism

Conventional fluid control is based on the use of valves. The valve, as an important component of the microfluidic chip, is used to block, split, and slow down the fluid. When a valve is used to block or slow down the fluid in a channel, the flow direction of the fluid may change. This allows us to control the fluid in the chip. To achieve a valve-like control effect on a single-layer chip, we propose a new control mechanism. The main idea of this proposal is to physically block, split, push, or slow down the fluid in a channel in a similar way as a valve, but without physical deformation of the channel. Due to the characteristics of the 3D-printed single-layer chip, we propose to block or slow down the flow of fluid in the channel by injecting air or gas directly into the channel. This induces a counter-pressure against the fluid’s flow direction.

By adjusting the pressure of the injected air or gas, fluid flow can be completely blocked, directed, divided, or slowed down. This method enables a single-layer chip to have valve-like fluid control without the use of valves. To implement this method, pressure injection holes (pressure inlets), identical to ordinary fluid inlets, are designed into the single-layer chip’s channel. It is important to note that the inlet hole can be positioned directly into the middle of the channel. At the same time, pressure release holes (pressure outlets) may be added to the design. Like the pressure inlet, the outlet can also be positioned directly into the middle of the channel. Depending on the application, both the pressure inlets and outlets need to be designed in the appropriate location. Fluid control is achieved by injecting or releasing appropriate pressure into or out of the in- and outlets using connected pressure or vacuum sources. However, a pressure outlet can also be standalone without a vacuum source, depending on the control requirement. When there is no pressure injected or released from the channel using the connected pressure or vacuum sources, the in- and outlets will not affect the fluid flow. While a peristaltic pump is not running, the not-moving rotor presses the tube inside the pump and the pump is closed, which means nothing can flow through the pump and the connected tube.

**Figure 2:**
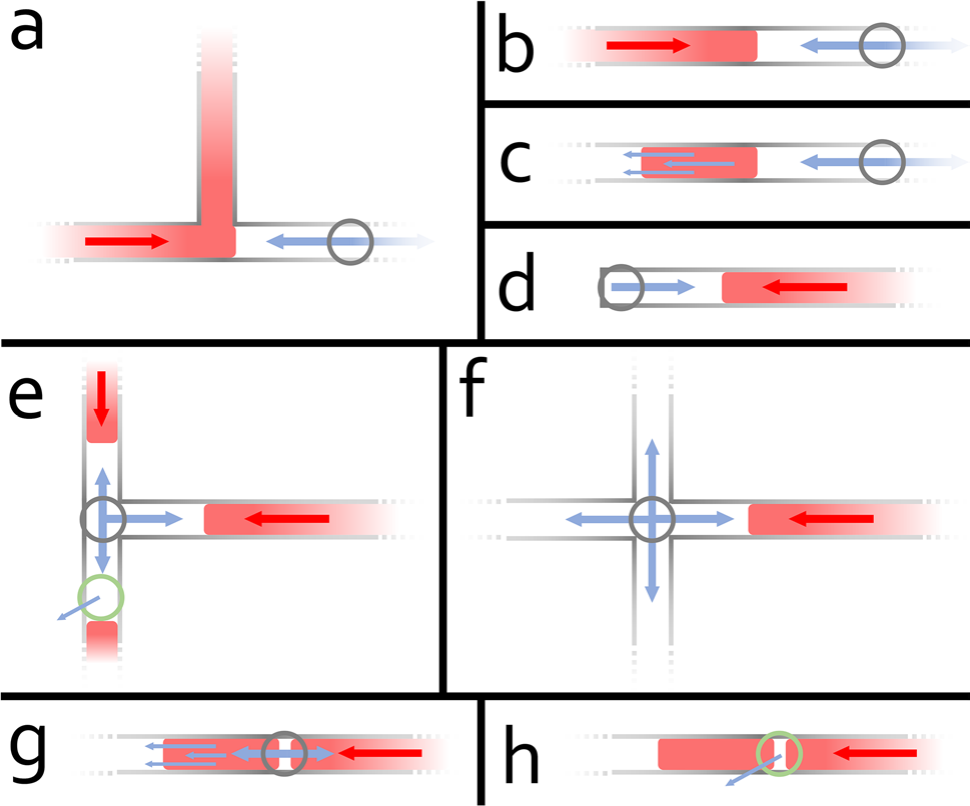
Illustration of possible usages of the presented active flow control mechanism on single-layer microfluidic chips. The red rectangle shape represents fluid in a channel. The red arrow represents where the fluid is coming from, and the blue arrow represents the added pressure and its direction. The grey circle represents the inlet of pressure, and the green circle the outlet. (**a**) A counter-pressure blocks the incoming fluid flow and only allows the fluid to flow to one channel (direction). (**b**) A counter-pressure blocks the incoming fluid flow (simple blockage). (**c**) The added pressure is pushing a segment of fluid content (transportation). (**d**) A counter-pressure slows down the incoming fluid flow in a dead-end channel (slow down). (**e**) A counter-pressure blocks the incoming fluid flows and stops the convergence of both fluids. An appropriate pressure outlet keeps other content in that channel from being affected. (**f**) Another example of fluid blockage in an intersecting channel. (**g**) A fluid sample flows through the pressure inlet point, and a counter-pressure is then added to block the fluid flow from further flowing and splits the fluid flow. A new free fluid segment is built (cut off). (**h**) The pressure outlet allows the release of air and lets the segment of fluid (re)merge with the main flow (merge).

Moreover, the design can be extended to further applications. Figure 2 shows some possible implementation scenarios in which this control mechanism can be designed into channels. Compared to control mechanisms using existing 3D-printed valve designs, our control mechanism offers notable advantages, particularly in the size of the microfluidic device, and no additional printing efforts are required.

**Figure 3:**
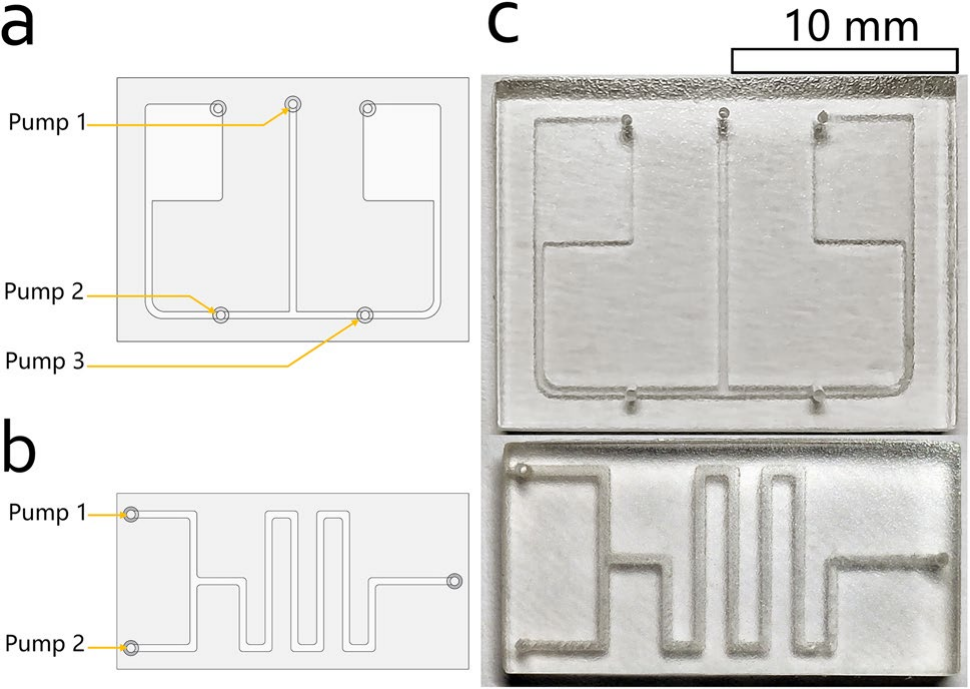
Single-layer 3D-printed microfluidic chips for demonstration of flow control using the system. (**a**) Design layout of the microfluidic chip (chip #1) for active flow control demonstration. This chip has one fluid inlet (top-center) connected to two on-chip reservoirs. On each reservoir is a fluid outlet (in the corner). Each connection channel has a pressure inlet for active control purposes (bottom left and right). The designed dimension of the chip is 15 $\times$ 20 $\times$ 4 mm (W $\times$ L $\times$ H) with a channel dimension of 400 $\times$ 800 µm (W $\times$ H). (**b**) Design layout of the microfluidic chip (chip #2) for passive mixing demonstration. This chip has two fluid inlets (left) connected to a serpentine formed channel, which has a fluid outlet at the end (right). The designed dimension of this chip is 10 $\times$ 20 $\times$ 4 mm (W $\times$ L $\times$ H) with a channel dimension of 400 $\times$ 800 µm (W $\times$ H). Connection setup for the experiments: (**a**) experiments #1 and #2—chip #1 is supplied with a colored sample by pump 1, while pump 2 and pump 3 control the fluid flow by adding pressure according to the program sequence; (**b**) experiment #3—chip #2 is supplied with a red-colored sample by pump 1 and green-colored sample by pump 2. The samples are mixed passively while flowing through the serpentine form channel. (**c**) Optical images of 3D-printed microfluidic chips (chip #1 and chip #2) using consumer-grade DLP 3D-printer.

### Experiments

To demonstrate this idea of active control, we designed a chip (chip #1, Fig. 3a) with one fluid inlet connected to two on-chip fluid reservoirs. Each connection channel has a pressure inlet. Two control sequences are used: (a) We direct the fluid coming from the fluid inlet to one reservoir at the T-junction using different control strategies, including (1) blockage and (2) direction of the fluid. (b) We split the fluid coming from the fluid inlet and push each segment to different reservoirs using different control strategies, including (1) split, cut off and (2) transportation. Further, we designed a conventional serpentine mixing device (chip #2, Fig. 3b) to demonstrate simple pressure-driven control. Figure 3c shows the two 3D-printed chips from above. We used the system to perform all the fluid control steps for every demonstrative experiment. We used food dyes colored water as fluid samples and stored the samples in the tubing. For the first experiment (experiment #1) using the chip #1, we programmed a sequence to perform it automatically, which is shown in Table 1.

**Table 1:** Program sequence for demonstrating active control handled by the system (experiment #1).

#	Pump name	Duration	Pressure (in %)
1	Pump 1	00:00:000–00:20:000	35
2	Pump 3	00:00:000–00:20:000	35

**Table 2:** Program sequence for demonstrating active control handled by the system (experiment #2).

#	Pump name	Duration	Pressure (in %)
1	Pump 1	00:00:000–00:20:000	45
2	Pump 2	00:15:000–00:30:000	55

**Figure 4:**
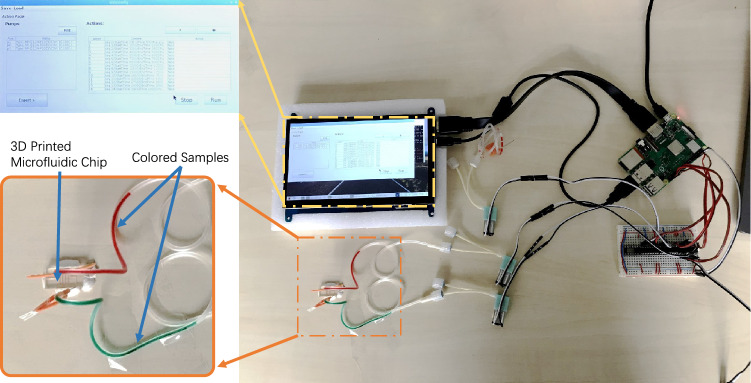
Picture of all parts of the all-in-one portable microfluidic system interconnected. All experiments are performed under this circumstance. The 3D-printed microfluidic is positioned on the table using sticky clay. The fluid samples are directly filled in the tubing. All operations are done via the touch screen.

**Figure 5:**
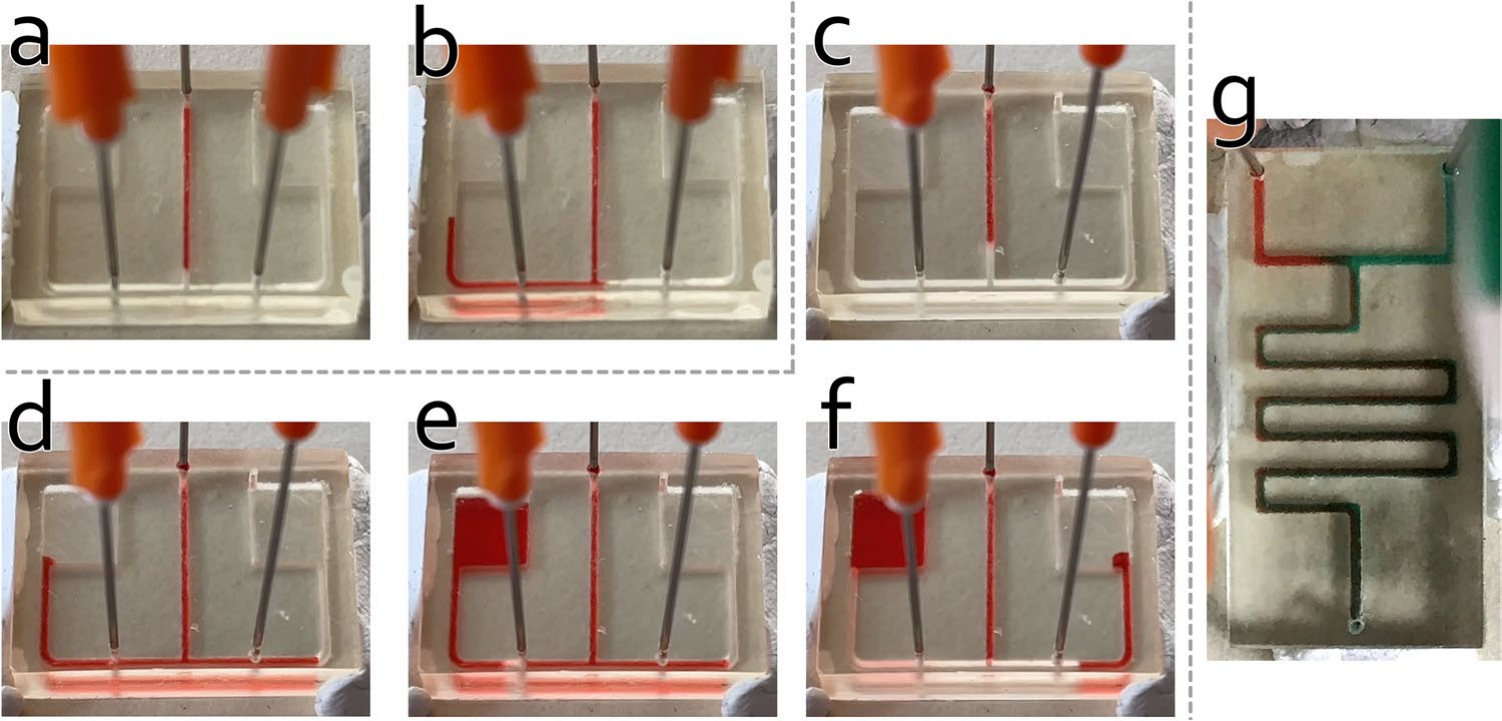
Pictures of the experiments. (**a**,**b**) Experiment #1 at different points in time: (**a**) fluid is supplying into the chip; (**b**) right pressure inlet points (pump 3) is supplied with air pressure. Hence right channel is blocked, fluid can only flow into the left channel. (**c**–**f**) Experiment #2 at different points in time: (**c**) fluid is supplying into the chip; (**d**) fluid flow into the left and right channel. Owing to the dimension variation of the channels, differences in hydraulic resistance happen, resulting different flow speed in left and right channels; (**e**,**f**) left pressure inlet points (pump 2) is supplied with air pressure. Hence fluid is split and transported to corresponding reservoir. (**g**) Picture of passive mixing (experiment #3) of two different colored samples using the system.

Briefly, the fluid inlet is supplying continuously with a red-colored fluid (sequence point #1), while one pressure inlet (right) is supplying with air to block the fluid flow. Here, we add pressure to the point at pump 3 (sequence point #2) to block the fluid flowing to the right, which will, therefore, direct the fluid flowing to the left reservoir. For the second experiment (experiment #2) using the chip #1, we also programmed a sequence, which is shown in Table 2. Briefly, the fluid inlet is supplying continuously with a red-colored fluid (sequence point #1), while 15 seconds later one pressure inlet (left) is supplying with air to split the fluid flow. Here, again only the fluid inlet is supplying the fluid. Fluid sample flows through the T-junction to both sides. After each connection channel is filled with fluid sample, we add pressure to the point at pump 2 (sequence point #2) to split and transport the fluid flow. The last experiment (experiment #3) demonstrates the conventional passive mixing experiment using chip #2. Table 3 shows the executed program sequence. Figure 4 shows how the system is used to perform the experiments. Figure 5a–f depict the experimental result of experiment #1 and experiment #2, and Fig. 5g depicts the experimental result of experiment #3.

**Table 3:** Program sequence for demonstrating conventional pressure-driven mixing experiment handled by the system (experiment #3).

#	Pump name	Duration	Pressure (in %)
1	Pump 1	00:00:000–00:30:000	35
2	Pump 2	00:00:000–00:30:000	35

**Table 4:** Total cost sheet for parts and materials used to prototype the system.

Product name	Quantity	Cost
Raspberry Pi 3 Model B+	1	€38.50
7-in. IPS Touchscreen	1	€48.99
Miniature Peristaltic RP-Q1.2N-P20Z-DC3V	4	€226.75
L293D	2	€2.40
Butterfly Winged Infusion Set 25G $\times$ 3/4”	4	€2.00
Accessory (cable, breadboard and power supply unit)	1	Ca. €20.00
Resin for one chip fabrication	1	Ca. €2.00
Total:		Ca. €340.00
Cost of the consumer-grade 3D-printer MiiCraft$^{\copyright }$ +		€4000.00

Some of the parts are used with an estimated value and indicated by “Ca.” The total cost of the entire system, including the material cost for chip fabrication, is about €340, which is relatively cheap compared to a conventional microfluidic system.

**Figure 6:**
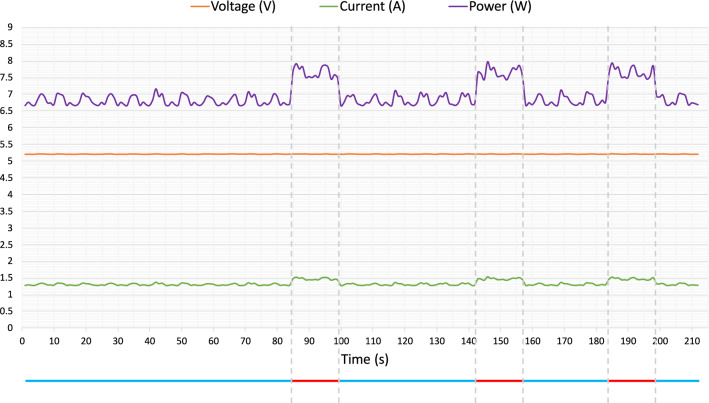
Power consumption of the system during idle and under full load. A digital multimeter was connected to the system’s power input to measure the voltage (V) and current (I) at a frequency of 1 Hz. Power consumption was then calculated as P = V $\times$ I. Vertical dashed lines indicate the starting and ending point of time of system load. Colored lines at the bottom of this figure indicate in which state the system was: blue—idle; red—full load. Full load refers to when all 4 pumps are running at 100%.

Power consumption is a critical performance metric of a portable system. We have measured the power consumption during idle and full load (simulated experiment where all 4 pumps are running at 100%). We used a digital multimeter (JT-UM25C, Joy-IT/Simac Electronics GmbH) to record the voltage and current of the entire system at a sampling rate of 1 Hz. The power consumption of the system in idle and full load was calculated by multiplying the current and the voltage (Fig. 6). The average power consumption during full load was 7.7 W, and during idle was 6.8 W. Even under full load, the power consumption is less than 1 W more than when the system is idling. This indicates the low power consumption of the pumps. The power consumption on average throughout an experiment is less than when the system is under full load. To enable use-on-the-go, a power bank or battery can be used to power the system. A typical power bank with a capacity of 10,000 mAh (32 Wh) can, therefore, last for ca. 4.5 h.

**Figure 7:**
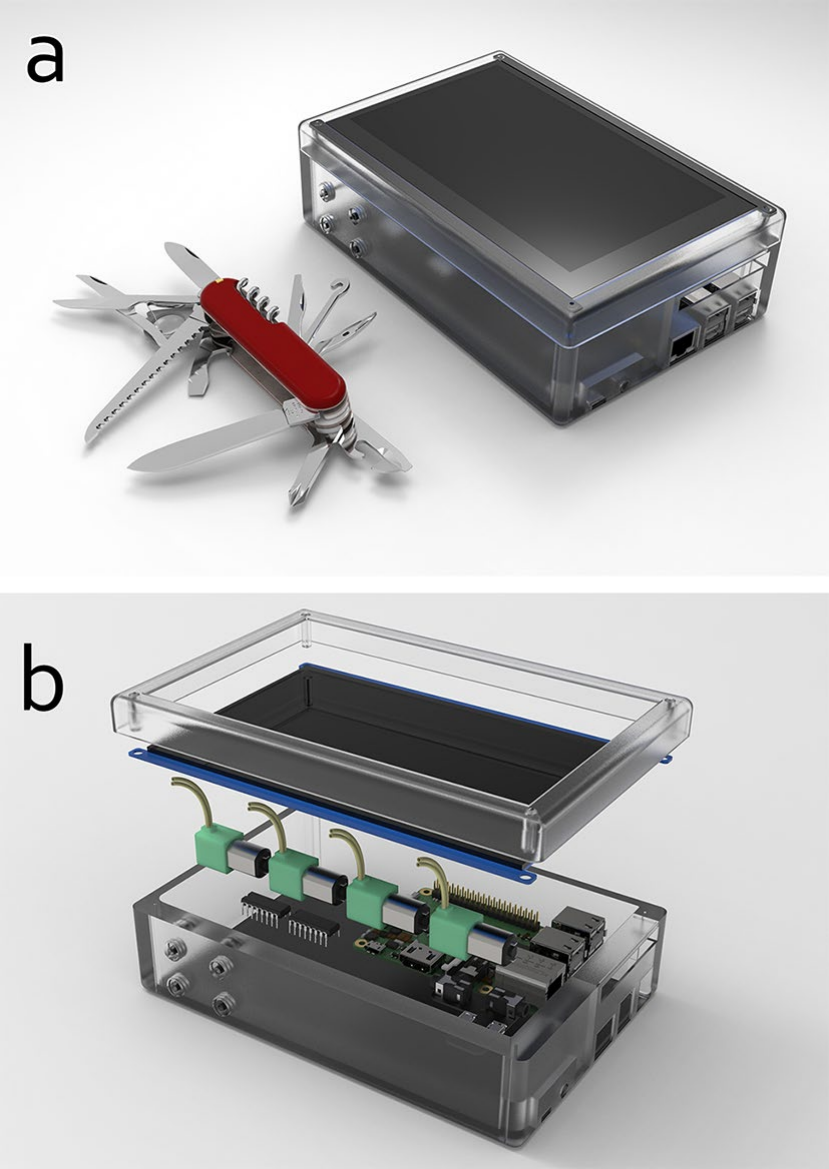
Render images of the 3D CAD model of the system with a plastic case. The system has a size of 12 $\times$ 18 $\times$ 5.5 cm (W $\times$ L $\times$ H). (**a**) A side-by-side size comparison of the system with a Swiss Army knife (91 mm model) in real-world size. (**b**) Exploded view showing the parts required for building the system, including a port extension board needed to use this case. The interconnections/cablings are not shown in this rendered image.

## Discussion

We demonstrated both active control, based on the proposed control mechanism, and simple pressure-driven automated fluid handling using the system on 3D-printed microfluidic devices. The programming and control were operated via the touchscreen of the system.

Further optimizations can be done on the system in the future. One optimization strategy is to add pressure reservoirs and loop-back pressure sensors in between. This can be achieved with ease. However, this will increase the size of the system. Moreover, adding reservoirs will slow down the response when changing the pressure. Another optimization strategy is to use another type of pump instead, such as a piezoelectric pump, which will increase the budget. We found the peristaltic pumps we are using gave an adequate performance for our experiments, but there is still room for improvement.

Improving the sealing will also improve the overall performance of the system. In this work, there was no special effort made to ensure the tight connection, as it was sufficient to demonstrate the experiments. However, it is desirable and feasible to improve the sealing in the future with better connections and components.

The system can connect up to 14 pumps directly to the GPIO ports and can connect more pumps by using a signal multiplexer in between. The system provides multiple connectivity options for further application possibilities, such as USB, Ethernet, Wi-Fi, Bluetooth, and GPIO. It is possible to connect devices such as a digital microscope or webcam to the system, allowing analysis and inspection of the experiment and the result directly on the system’s screen. Integrating additional sensors or control devices into the system is also possible and enables integrated analysis and sensor data based flow control.

The system proposed here is based on a fully-functional tiny computer, and thus, many applications can be performed directly on the system. The entire cost of the control system is listed in Table 4. The relatively low cost and accessible parts make the system suitable for educational purposes. This allows students to design experiments and use this system to perform them with the self-3D-printed microfluidic chip.

We have modeled a case for the system with a dimension of 18 $\times$ 12 $\times$ 5.5 cm (Fig. 7), which can contain all components and provides further space for additional pumps or devices. The total weight without the case is 446 g. Although we did not fabricate the case, the estimated total weight, including such a plastic case, is 600 g. The compactness of the system can be further improved by choosing smaller components such as a 5-in. or 3.5-in. touchscreen. This can also further reduce the size, weight, and power consumption of the entire system.

The entire system is built with low-cost, accessible parts and uses a 3D-printed microfluidic device. We introduced a way of controlling fluid in a single-layer 3D-printed microfluidic device without implementing any existing valve designs and demonstrated the feasibility. The system gives scientists and developers further application possibilities by extending the hardware and software. We believe this portable all-in-one microfluidic system and our proposed control mechanism are enabling technologies. The low cost and accessible parts can help spread microfluidics, especially in the DIY community and education. This system can find broad applications, especially in point-of-care medical diagnostics, environmental pollution testing, food safety inspection, biohazard detection, and biological research. Integrating sensors and cameras into the system may one day enable the realization of compact and mobile handheld diagnosis and analysis devices for different kinds of medical usage.

## Methods

Pamicon consists of accessible, low-cost and commercially available parts. Table 4 summarizes the parts and detailed costs. We describe the critical points of Pamicon in detail in the following sections.

### Single board computer

A single board computer provides a full-fledged computer experience. Hence, we do not need an extra host computer to operate the system. The Raspberry Pi is very popular in the DIY community and runs a desktop-grade Linux operating system. Besides, it provides 40-pin GPIO (general-purpose input/output) ports. We used the Raspberry Pi 3 B+ for this work, along with a 64 GB MicroSD card, which contains the Raspbian Linux system and our user interface software. The pumping system is connected to the SBC via GPIO ports. The control of the pumping system is handled by our software and runs on top of the Linux system. The SBC sends digital signals via GPIO ports to the pumping system to control it. A 7-in. touchscreen is connected to the SBC, with which the user can operate the microfluidic system. The SBC is powered by a 5 V power supply unit, which can be switched to a battery or a power bank. This power supply unit is the only power input for the entire system. The pumping system acquires power via the 5 V GPIO output port of the SBC.

### Pumping system

The pumping system is designed to generate variable pressure according to the user’s needs. We used four peristaltic ring-type miniature pumps (RP-Q1.2N-P20Z-DC3V, Aquatech Co. Ltd/ Takasago Electric, Inc.). The number of pumps can be extended, and individual pumps can be changed to other models according to the need. The pump model we used can generate both positive and negative pressure up to ± 7.3 psi and has a rated input voltage up to 5 V. The pumps are connected to the driver (signal amplifier) ICs (L293D, Texas Instruments). The L293D is designed to provide bidirectional drive currents of up to 600 mA at voltages from 4.5 to 36 V. Each L293D IC can drive up to two pumps for bidirectional setup. This means, depending on the signal (polarity), each pump can both charge (positive pressure) or discharge (negative pressure) the content. The driver ICs are connected to the SBC and amplify the signal from the connected GPIO ports. To drive the pumps, the 5 V pin from SBC’s GPIO is connected to the driver ICs. The incoming digital signal controls whether the pump should run or not, and by using the PWM (pulse-width modulation) method, the pressure of each pump can be regulated. On each end of a pump, a male Luer-Lok fitting is connected for an easy swap of tubing. The pumping system in our microfluidic system is designed without pressure reservoirs, and the tubing can be connected directly to the microfluidic chip. Depending on the application, it can be extended with pressure reservoirs in between.

### Control software

A control software with an intuitive GUI (graphical user interface) was implemented to allow user control of the system. We designed the software to provide a comfortable, straightforward user experience by enabling both manual and automated control of the system. As for automated control, the user can program the sequence of operations which will be executed by the software. The programmed sequence is based on a timeline and executed chronologically. Each operation is defined on the timeline by (1) selecting a connected pump, (2) the pressure level, (3) the flow direction (pressure or vacuum), and (4) the start and end time of the operation. At any point in time, there can be multiple operations running simultaneously, and each operation is running independently. Hence, each operation can have an individual pressure level and a different flow direction. Since the SBC provides many GPIO ports, the user can add and predefine pump configurations along with the connection pin and some model-specific fine-tuning settings on the software GUI, which can be selected during programming. For convenience, the sequence can be saved for future reuse and modification. As for manual control, each pump can be operated with an individual setting. One significant advantage of using an SBC as the logic board is that the user can extend the application by writing his specific software directly on our system in familiar programming languages such as Python, Scratch, Processing, C/C++, or Java. All of these languages support the access of the GPIO ports^23–25^. Thus, with the many connection ports available on the board, the user can extend the application easily by including other devices such as sensors or different pumps and control them using his own software.

### Chip design and fabrication

We used the Miicraft$^{\copyright }$ + DLP 3D-printer to fabricate the designed experiment chips. We first modeled the chips using CAD 3D modeling software, then sliced the designs into layers of 50 µm with the software accompanying the 3D-printer. Every chip is printed with a layer exposure time of 7 s. As an exception, the first two layers are exposed for 60 s each. The printing speed was set to “Slow”. The chip #1 was designed as baseless, which means it was laminated after the printing process, whereas the chip #2 was directly printed as a unibody, which means the channels are sealed printed into the chip. As the post-curing process, both chips were further exposed in a UV chamber for 2 h after the printing process. We used an accessible medical infusion set to connect the chip with the system.

## Data Availability

In this work implemented microfluidic control software, chip designs and the case model are available on the author’s website: https://z-y.download/?dir=/Pamicon.
